# Oscillations of the p53-Akt Network: Implications on Cell Survival and Death

**DOI:** 10.1371/journal.pone.0004407

**Published:** 2009-02-06

**Authors:** Keng Boon Wee, Uttam Surana, Baltazar D. Aguda

**Affiliations:** 1 Institute of Molecular and Cell Biology, A*STAR (Agency for Science, Technology and Research), Proteos, Singapore; 2 Mathematical Biosciences Institute, The Ohio State University, Columbus, Ohio, United States of America; 3 Center for Critical Care, OSU Medical Center, The Ohio State University, Columbus, Ohio, United States of America; National Institutes of Health, United States of America

## Abstract

Intracellular protein levels of p53 and MDM2 have been shown to oscillate in response to ionizing radiation (IR), but the physiological significance of these oscillations remains unclear. The p53-MDM2 negative feedback loop – the putative cause of the oscillations – is embedded in a network involving a mutual antagonism (or positive feedback loop) between p53 and AKT. We have shown earlier that this p53-AKT network predicts an all-or-none switching behavior between a pro-survival cellular state (low p53 and high AKT levels) and a pro-apoptotic state (high p53 and low AKT levels). Here, we show that upon exposure to IR, the p53-AKT network can also reproduce the experimentally observed p53 and MDM2 oscillations. The present work is based on the hypothesis that the physiological significance of the experimentally observed oscillations could be found in their role in regulating the switching behavior of the p53-AKT network between pro-survival and pro-apoptotic states. It is shown here that these oscillations are associated with a significant decrease in the threshold level of IR at which switching from a pro-survival to a pro-apoptotic state occurs. Moreover, oscillations in p53 protein levels induce higher levels of expression of p53-target genes compared to non-oscillatory p53, and thus influence cell-fate decisions between cell cycle arrest/DNA damage repair versus apoptosis.

## Introduction

In response to DNA damage from exposure to ultraviolet (UV) or ionizing radiation (IR), damped oscillations in the levels of p53 and MDM2 proteins have been observed in populations of cells such as fibroblasts (human and mouse) and MCF7 breast cancer cells [1], [2]. Isolated single cells exhibit oscillations that are more sustained, sometimes lasting more than three days, depending on the persistence of DNA damage [3]. The frequencies of these oscillations generally increase with increasing IR intensity, and oscillation periods are typically in the range of 4 to 7 hrs; unlike this narrow range in the observed periods, the amplitudes of p53 oscillations are quite variable. Because it is the number of oscillations rather than their amplitudes that is observed to be dependent on the dose of IR, p53 oscillations have been described as ‘digital’. MDM2 oscillations are coupled with the p53 oscillations, with corresponding peaks separated by 1.5 to 2.5 hrs. Similar observations have also been observed *in vivo* in mouse intestine and spleen [4] where damped p53 pulses with periods of 4.5 to 6 hrs are reported.

The putative mechanistic origin of the oscillations is the negative feedback loop between p53 and MDM2 [1], [3], [5]–[9]. Transcriptional activity of p53 induces MDM2 expression [10], and MDM2 ubiquitinates p53 thereby marking the latter for degradation [11]–[17]. DNA damage, such as double strand breaks (DSBs), triggers a cascade of intracellular signaling pathways that lead to post-translational modifications (e.g., phosphorylation) of both p53 and MDM2, rendering p53 more stable with enhanced transcriptional activity [18]–[20] and MDM2 less stable with shortened half life [21]. The net result is that DNA damage-induced protein modifications prolong the time for MDM2 to cause p53 degradation; this time delay is necessary to generate p53-MDM2 oscillations [1], [3], [6], [9].

**Figure 1:**
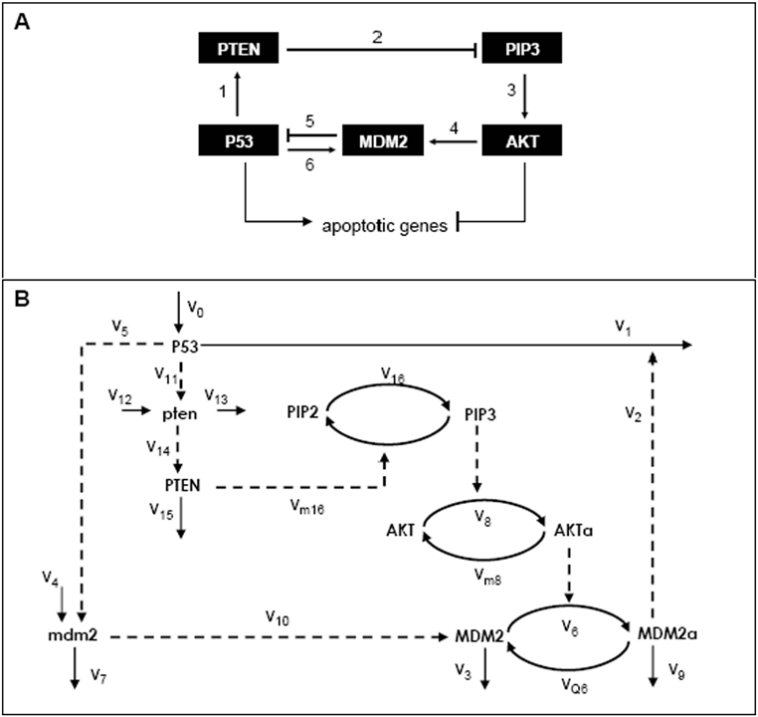
(A) Qualitative network showing mutual antagonism between AKT and p53. An arrow means a pathway that leads to activation or upregulation; a hammerhead represents inhibition or downregulation. AKT is antagonized by p53 via edges 1–3, and p53 is antagonized by AKT via edges 4–5. A p53-MDM2 negative feedback loop is shown by edges 5 and 6. (B) The oscillatory p53-AKT model (the *Model*). The rate expressions v_i_'s are given in Table 1. Note: mdm2 and pten denote mRNAs of MDM2 and PTEN gene, respectively. Mechanisms of the p53-AKT mutual antagonism: the network is activated by two cellular events namely growth factor stimulation and cellular stress. The former leads to phosphorylation of PIP2 (phosphatidyl inositol-4,5-bisphosphate) to PIP3 (Phosphatidyl inositol 3,4,5. triphosphate) and thereby PIP3-mediated activation of AKT [43]. Active AKT (AKT*a*) phosphorylates MDM2 leading to MDM2*a* translocation to the nucleus [44] where it inhibits p53, and thereby promotes cell survival [45]–[50]. Under cellular stress however, transcriptionally active p53 induces PTEN [51]–[54] among other target genes [55]. PTEN antagonizes AKT-mediated cell survival by deactivating PIP3 through efficient dephosphorylation [56]. p53-dependent transcription of PTEN upon irradiation-induced DNA damage has been reported in MCF-7 (human breast cancer epithelial), A172 (human glioblastoma), MEF (mouse embryonic fibroblasts) and tissues of mouse small intestine, colon, kidney and liver [57]–[59] where p53 oscillations are also observed [1], [3]–[5], [60].

The p53-MDM2 loop is, of course, not isolated from other molecular interactions in a cell. For example, as depicted in Figure 1A, the p53-MDM2 loop is embedded in a larger network that includes AKT, a gene associated with cell survival signaling pathways. (There are several isoforms of AKT, but among these, we will specifically refer to AKT1 as AKT in this paper). The significance of this p53-AKT network (Figure 1A) is highlighted by the following facts: it involves two known tumor suppressor genes (p53 and PTEN) [22]–[26], and two oncogenes (MDM2 and AKT) [27]–[29]. Note that the p53-AKT network is composed of two feedback loops: the mutual antagonism between p53 and AKT which is a positive feedback loop (edges 1 to 5), and the p53-MDM2 negative loop (edges 5 and 6). The mutual antagonism between p53 and AKT is significant because p53 and AKT have opposite effects on apoptosis. p53 induces the expression of pro-apoptotic genes such as PUMA, BAX, NOXA, FAS and BAD [30]–[34], while AKT inhibits (by phosphorylating) pro-apoptotic proteins such as BAD [35], [36], CASPASE-9 [37] and FKHRL1 [38]. Note that BAD is a common target of p53 and AKT [39], which may further explain BAD's crucial role in apoptosis [40]. These considerations led us to hypothesize that the p53-AKT network is an important module that controls pathways to cell survival or death [41], [42].

The major issue addressed in this paper is the impact of the oscillatory dynamics of the p53-MDM2 loop on the antagonism between pro-apoptotic pathways (promoted by high p53 levels) and pro-survival pathways (promoted by high AKT actvity). We have shown earlier [41] that the p53-AKT network predicts an all-or-none (bistable) switching behavior between pro-survival and pro-apoptotic states, and will now show how this switch is affected by p53-MDM2 oscillations.

## Results

### Model Formulation

The model mechanism considered here is shown in Figure 1B; it will be referred to from hereon as the *Model*. It is an extension of a model we previously studied (model Q3 in Ref. [41]) which exhibits a bistable switch between pro-survival and pro-apoptotic cellular states. The said model extension involves two additional variables, namely, the mRNA transcripts of the *MDM2* and *PTEN* genes; these transcripts are symbolized by mdm2 and pten, respectively, in Figure 1B. The inclusion of mdm2 introduces a time delay in the negative feedback loop between p53 and MDM2 proteins, and increases the potential for oscillations. The dynamics of the *Model* is described by the set of ordinary differential equations (ODEs) and parameter values that are listed in Table 1.

**Table 1:** Kinetic rate equations and parameters of the *Model*.

Differential Equations			Kinetic Parameters		
			Chosen values		Range
*d[p53]/dt*	* = *	*v_0_ - v_1_ - v_2_*	*k_0,basal_ = 0.02*		*0.005–0.2*
*d[AKTa]/dt*	* = *	*v_8_ - v_m8_*	*k_d_ = 0.02*		*0.02–0.2*
*d[MDM2a]/dt*	* = *	*v_6_ - v_Q6_ - v_9_*	*k_2_ = 0.0625*		*0.0184–0.092*
*d[MDM2]/dt*	* = *	*v_10_ - v_3_ - v_6_+v_Q6_*	*j_2_ = 0.01*		*0.03–0.3*
*d[mdm2]/dt*	* = *	*v_4_+v_5_ - v_7_*	*k_5_ = 0.0375*		*0.024*
*d[PTEN]/dt*	* = *	*v_14_ - v_15_*	*j_5_ = 0.5*		*∼1*
*d[pten]/dt*	* = *	*v_11_+v_12_ - v_13_*	*k_6_ = 22*		*22* (Ref. [81])
*d[PIP3]/dt*	* = *	*v_16_ - v_m16_*	*j_6_ = 0.6*		*0.5±0.1* (Ref. [81])
			*k_Q6_ = 0.5*		*0.0000297–2.92*
*[AKT_Tot]*	* = *	*[AKT]+[AKTa]*	*j_Q6_ = 0.1*		*0.00238–2.23*
*[PIP_Tot]*	* = *	*[PIP2]+[PIP3]*	*k_8_ = 20*		*∼20*
			*j_8_ = 0.1*		*∼0.1*
*v_0_*	* = *	*k_0_*	*k_m8_ = 0.2*		*0.0000297–2.92*
*v_1_*	* = *	k_d_ * [p53]	*j_m8_ = 0.1*		*∼0.1*
*v_2_*	* = *	k_2_ * [MDM2a] * [p53]/(j_2_+[p53])	*k_10_ = 0.02*	*(New)*	(Ref. [6])
*v_3_*	* = *	d_MDM2_ * [MDM2]	*p_mdm2_ = 0.0009*	*(New)*	(Ref. [6])
*v_4_*	* = *	*p_mdm2_*	*d_mdm2_ = 0.01*		(Ref. [6])
*v_5_*	* = *	k_5_ * [p53]^n2^/(j_5_^n2^+[p53]^n2^)	*d_MDM2_ = 0.005*		*0.0028–0.0347*
*v_6_*	* = *	k_6_ * [AKTa] * [MDM2]/(j_6_+[MDM2])	*d_MDM2a,basal_ = 0.005*		*0.0028–0.0347*
*v_Q6_*	* = *	k_Q6_ * [MDM2a]/(j_Q6_+[MDM2a])	*k_11_ = 0.006*		*∼0.006*
*v_7_*	* = *	d_mdm2_ * [mdm2]	*j_11_ = 2*		*>1*
*v_8_*	* = *	k_8_ * [PIP3] * [AKT]/(j_8_+[AKT])	*d_PTEN_ = 0.0054*		*0.0025–0.0083*
*v_m8_*	* = *	k_m8_ * [AKTa]/(j_m8_+[AKTa])	*p_pten_ = 0.0009*	*(New)*	*–*
*v_9_*	* = *	d_MDM2a_ * [MDM2a]	*d_pten_ = 0.01*	*(New)*	*–*
*v_10_*	* = *	k_10_ * [mdm2]	*k_14_ = 0.02*		*–*
*v_11_*	* = *	k_11_ * [p53]^n1^/(j_11_^n1^+[p53]^n1^)	*k_16_ = 0.15*		*∼0.15*
*v_12_*	* = *	*p_pten_*	*j_16_ = 0.1*		*∼0.1*
*v_13_*	* = *	d_pten_ * [pten]	*k_m16_ = 73*		*73±4.4*
*v_14_*	* = *	k_14_ * [pten]	*j_m16_ = 0.5*		*0.1–1*
*v_15_*	* = *	d_PTEN_ * [PTEN]	*n_1_, n_2_ = 4*		
*v_16_*	* = *	k_16_ * [PIP2]/(j_16_+[PIP2])	*k_0, IR_ = 0.0025*	*(New)*	
*v_m16_*	* = *	k_m16_ * [PTEN] * [PIP3]/(j_m16_+[PIP3])	*d_MDM2a,IR_ = 0.0025*	*(New)*	
			*[AKT_Tot] = 1*		
*Note:*			*[PIP_Tot] = 1*		
*k_0_*	* = *	k_0,basal_+k_0,IR_ *ρ * exp(-λ*t)			
*d_MDM2a_*	* = *	d_MDM2a,basal_+d_MDM2a,IR_ *ρ * exp(-λ*t)			

New kinetic rate equations and parameters (relative to those used in Ref. [41]) are indicated. Time is in *minutes*; concentration is in *µM*.

Post-translational modifications of p53 and MDM2 due to DNA damage are assumed to be reflected in an increase of the rate parameter *k_0_* (for p53 synthesis and activation) and of the rate parameter *d_MDM2a_* (for degradation of active MDM2). Another assumption made is that *k_0_* and *d_MDM2a_* are directly proportional to IR intensity (*ρ*), that is, k_0_ = k_0,basal_+k_0,IR_*ρ and d_MDM2a_ = d_MDM2a,basal_+d_MDM2a,IR_*ρ. The justification of this assumption is based on the many known pathways associated with IR-induced DNA damage that upregulate p53 and down-regulate MDM2 (see Figure S1 of *Supplemental Data* for details).

### Steady States and Oscillations

**Figure 2:**
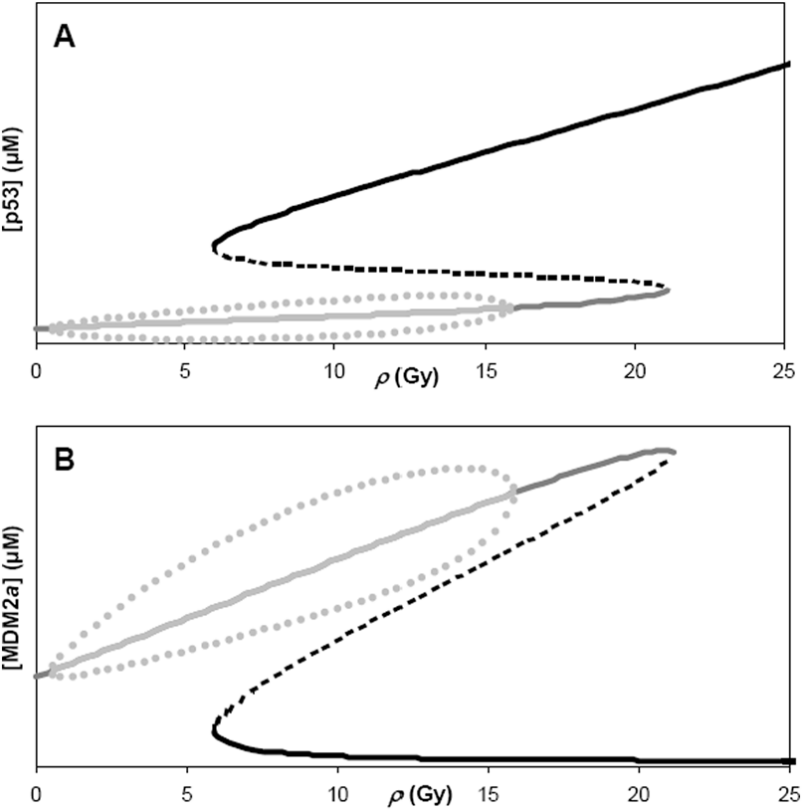
Steady states of (A) p53 and (B) active MDM2 (MDM2*a*) as a function of the intensity of ionizing radiation, *ρ*. Local stability of these states is indicated as either stable (black curve: stable nodes; dark gray curve: stable spirals) or unstable (broken black curve: saddle points; light gray curve: limit cycles). Limit cycle oscillations whose amplitudes are shown by the dots arise from the unstable steady states (light gray curve) in the lower (A) or upper (B) branch of the steady state curves.

The steady states of the *Model* are determined by equating all the right-hand sides of the ODEs in Table 1 to zero, and solving for the roots of the resulting system of algebraic equations (see *Materials and Methods*). The steady states of p53 and active MDM2 (MDM2*a*) as functions of *ρ* are shown in Figure 2. The *Model* exhibits multiplicity of steady states within a range of *ρ* (6 to 20.8 Gy – in Figure 2). Within this range of *ρ*, the high-p53 (correspondingly, low-MDM2*a*) steady states are all locally stable nodes, and the middle branch of steady states are all unstable saddle points. Decreasing from the right-knee of the steady state curves to *ρ* ∼16 Gy, the dynamics about the low-p53 (high-Mdm2a) branch of steady states is damped oscillatory (stable spiral). The steady states enveloped by the gray dotted curves in Figure 2 exhibit unstable spirals that lead to sustained oscillations (limit cycles). The peaks and troughs of these periodic oscillations are indicated by the dots above and below the steady states, respectively. It is interesting to note that the steady state at *ρ* = 0 Gy is a stable spiral (damped oscillations); this is reminiscent of reported experimental observations of damped oscillations in some cell populations that are not exposed to DNA damage-causing radiation [3].

Generally, we found that the frequencies of the oscillations in p53, mdm2, MDM2 and MDM2*a* increase with increasing *ρ* (Figure S2 of *Supplemental Data*), in accord with experimental observations [3]. Furthermore, the oscillation frequencies of p53, mdm2, MDM2 and MDM2*a* are identical. The *Model* predicts oscillation periods of 3.5 to 5.2 hours, well within the reported range of experimental values [3].

The *Model* also reproduces experimental measurements of the time separation or delay (1.2–1.9 hrs) between the peaks of MDM2*a* and p53 oscillations. Generally, these time delays are not sensitive to *ρ* (Figure S2 of the *Supplemental Data*). The *Model* also predicts that the amplitudes of the oscillations of active AKT, PIP3, pten mRNA and PTEN will be difficult to measure experimentally because the concentration differences between crests and troughs are less than 4% of the mean concentration (data not shown) – this could explain why oscillations in these molecules have not been reported so far.

### Cells exposed to increasing IR intensities

**Figure 3:**
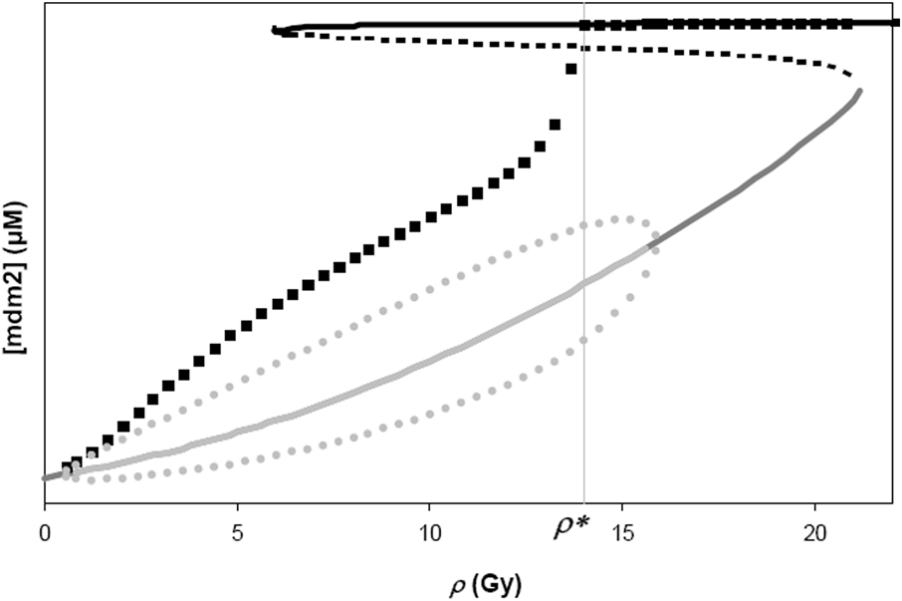
The steady-state curve of mdm2 mRNA is plotted as a function of the intensity of ionizing radiation, *ρ*. The black squares indicate the first peaks of the mdm2 oscillations when the *Model* is simulated at different values of *ρ*, with each simulation having identical initial conditions (equal to the steady states of the system at *ρ*  =  0 Gy). The vertical line at *ρ* = ρ* = 13.8 Gy marks the value where the peak of mdm2 oscillation is attracted to the upper branch of steady states.

Computer experiments using the *Model* were performed to simulate the behavior of a cell that is exposed to a pulse of IR with fixed intensity *ρ*. For each computer simulation, the initial cellular levels of the proteins and transcripts are those of the steady states of a cell that is not exposed to IR (that is, the steady state of the *Model* with *ρ* = 0 Gy). As we increase *ρ* in the range where limit cycle oscillations are exhibited, the system initially displays a high-amplitude oscillation that settles down to the unique limit cycle with smaller amplitude at each *ρ*. Initially large amplitude p53 oscillations are observed in experiments with single cells [3]. The peaks of these initial oscillations are shown as black squares in Figure 3, and superimposed with the steady-state curve of mdm2. This figure shows that the initial peaks increase with *ρ* and that there is a particular *ρ* (symbolized by ρ*) where the system crosses a boundary surface associated with the unstable steady states (the dotted middle branch of steady states) and gets attracted to the upper branch of stable steady states. Since ρ* is less than the value of *ρ* corresponding to the right-knee of the steady state curve, we refer to ρ* as an *early-switching point*.

### Effect of cell-cell variation

**Figure 4:**
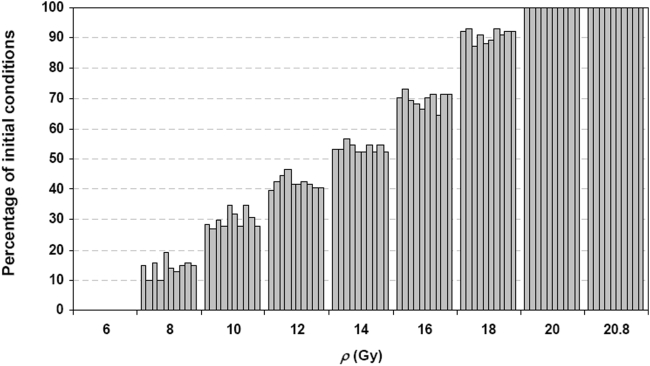
Percentage of Latin sets of initial concentrations leading to high-p53 states. The initial concentrations of all molecular species are varied simultaneously, with deviations ranging from 0 up to 200% of the steady state concentration at *ρ  = * 0 Gy (range is divided equally into 100 intervals). Hypercube sampling, which involves random permutations of these intervals, is used to select 101 unique Latin sets of initial conditions. The percentage of these sets of initial conditions leading to high-p53 state at various selected *ρ* is computed. A total of 10 independent hypercube samplings are performed and the result of each hypercube sample is shown in the figure as a bar.

In our computer simulations, cell-to-cell variability is reflected in the distribution of initial concentrations of all the molecular species in the *Model*. For every molecular species, the initial concentration is varied around its steady state concentration at *ρ* = 0 Gy, with deviations ranging from 0 up to 200% of the steady state (this range is divided equally into 100 intervals and then randomly permutated to generate 101 Latin sets of initial conditions in each Latin hypercube sample). A total of 10 independent Latin hypercube samplings are carried out. For each Latin set of initial concentrations, trajectories are computed for each *ρ* within the range where there are multiple steady states (6 to 20.8 Gy). The percentages of sets of initial concentrations that lead to high-p53 steady states are given in Figure 4. Note that 10–20% of initial conditions can lead to an early-switching point at *ρ* equal to 8 Gy. At 18 Gy about 90% of the cells are predicted by the *Model* to make this transition. Thus, switching from the oscillatory state to the high-p53 state can be induced by cell-cell variations in their initial concentrations at *ρ* that are significantly lower than that corresponding to the right-knee of the steady-state curve (see Figure 2). Nevertheless, the propensity for early switching to high-p53 states becomes more deterministic (less dependent on cell-cell variations) as *ρ* increases.

### Effect of cell-type variation

Measured basal concentrations of p53 in seven different cell lines range from about 1×10^4^ to 22×10^4^ molecules per cell [6], suggesting a cell type-dependent rate of p53 synthesis, and therefore a significant variation in the value of the parameter *k_0,basal_* of the *Model*. In addition, several reports [61]–[63] have documented cell-type specific post-translational modifications of various DNA-binding domains of p53 – suggesting that the model parameter *j_5_* (associated with p53's affinity to the promoters of target genes) is also subject to wide variations depending on cell types. We investigated how these cell-type specific differences affect the behavior of the system by varying the parameters *k_0,basal_* and *j_5_*. For each combination of *k_0,basal_* and *j_5_*, a steady-state curve is computed. We sampled 4896 combinations of *k_0,basal_* and *j_5_* , and determined their corresponding steady-state curves. All steady-state curves with multiple steady states exhibit either limit cycles or damped oscillations, or both, at the low-p53 steady state branch. Altogether, three general types of bifurcation curves or switching behavior are obtained among the 4896 parameter combinations (Figure S3 of the *Supplemental Data)*:

1. Monostable (observed in 9% of the steady state curves). The only state of the system is a high-p53 stable node and no oscillation is manifested. This occurs at relatively high p53 production rate and fast dissociation rate of p53 from promoter site.
2. Early switch (observed in 87% of the steady state curves). This is the predominant type observed.
3. Saddle-node switch (observed in 4% of the steady state curves). For this type, the system switches to high-p53 state exactly at the right-knee of the curve.

Thus, the early switching phenomenon is likely to arise in cell types that exhibit p53 oscillations.

### Consequences of p53 oscillations on expression of target genes

Since p53 is a transcription factor with many target genes [64], it is of interest to determine what the effects of p53 oscillations are on the expression levels of these genes. As an example, we considered a simplistic model for the expression of a representative p53-target gene X. This model includes the rate equations v_11_, v_13_, v_14_ and v_15_ shown in Figure 1B. The time-course of an oscillatory p53 is estimated by 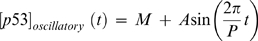, where *M* is the mean p53 level between the crest and trough, *A* is the oscillation amplitude, and *P* is the oscillation period. The values of the parameters *M*, *A*, and *P* depend on *ρ*, and these values are estimated from the oscillations generated in Figure 2. The other rate expressions and all the kinetic parameters of this simplistic model are identical to those used in the *Model* (Figure 1B).

**Figure 5:**
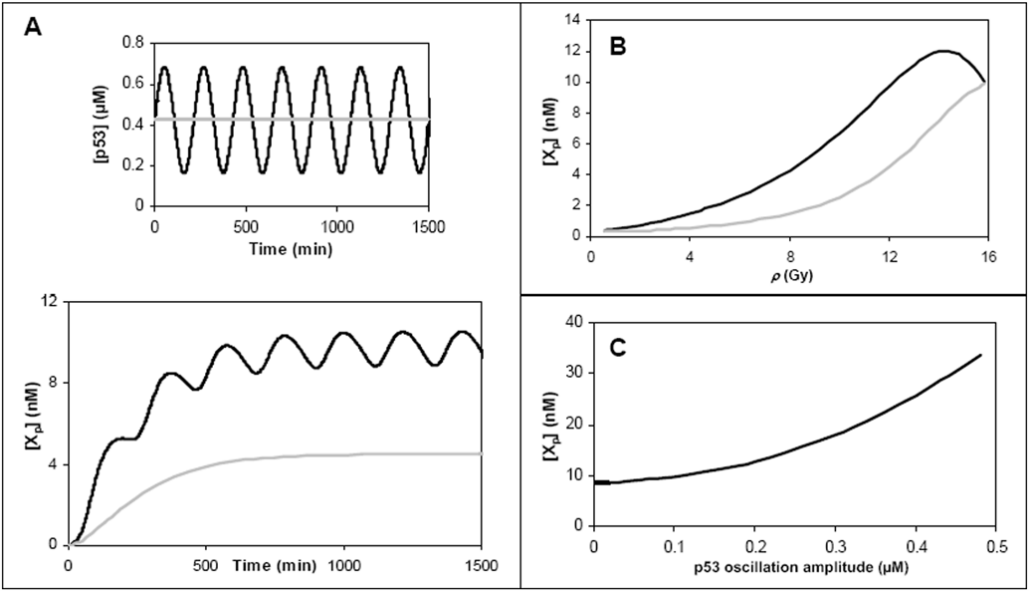
Consequences of p53 oscillations on gene expression. (A) Time courses of X_p_ expression induced by both non-oscillatory (gray) and oscillatory p53 (black) shown in the upper panel (this oscillation profile of p53 is similar to the limit cycle of the *Model* at *ρ*  = 12Gy). The level of the non-oscillatory p53 is equal to the mean of the oscillating values of p53. (B) Steady state level of X_p_ induced by both non-oscillating (gray curve) and oscillating (black curve) p53 for the entire range of *ρ* where limit cycle exists in the *Model*. The black curve is obtained by taking the average of the crest and trough of X_p_ at steady state. Similarly, the oscillation profiles of p53 at each *ρ* are estimated from the limit cycle of the *Model*. The level of non-oscillatory p53 is equal to the mean of the oscillatory p53. (C) Steady state level of X_p_ induced as a function of p53 oscillation amplitude. Parameter values used in the simulations (see text): the mean (*M*) and period (*P*) of oscillatory p53 are fixed at 0.5 µM and 4 hr, respectively. The trend shown in (C) is similar to those obtained using other values of p53 oscillation amplitudes (*A* = 0 to 0.5 µM), means (*M* = 0.1 to 2 µM) and periods (*P* = 4 to 7 hrs).

Figure 5A shows examples of temporal profiles of X_p_ (protein of p53-target gene X) expression for both non-oscillatory and oscillatory p53. As clearly shown, the rate of X_p_ synthesis is significantly increased when p53 oscillates. Figure 5B shows that the steady state of X_p_ is increased due to p53 oscillations for the entire range of *ρ* where limit cycles are manifested, despite the fact that the mean level of p53 oscillations is equal to that of non-oscillatory p53. We observe that the steady state level of X_p_ depends, not on the period *P* of p53 oscillations (data not shown), but on the mean (*M*) and amplitude (*A*) of the oscillations. The steady state levels of X_p_ increase with *A*, as depicted in Figure 5C.

Many examples of the target protein X_p_ can be cited from the literature. For example, the cyclin-dependent kinase inhibitor p21 is a transcriptional target of p53 and has been observed to oscillate in response to p53-MDM2 oscillations upon irradiation in a wild-type human cell population [2]. In addition, cDNA microarray data analysis of p53 target genes revealed 51 genes whose mRNA levels oscillate [65].

To investigate whether p53 oscillations could also induce higher levels of expression of target genes with slow expression kinetics, simulations similar to Figure 5B are performed for each of the following four cases: (i) p53 binding affinity to gene promoter is decreased 2.5 fold, (ii) p53 induction of genes occurs 8 hrs post-irradiation, (iii) p53 transcriptional rate is decreased 6 fold, and (iv) target protein degradation rate is decreased 4 fold. Case (i) assumes that p53 binding to a promoter affects the timing of gene transcription. Lower p53 binding affinity to promoters has been invoked by others [66], [67] to explain the rapid induction of cell cycle genes (within 2 hrs) compared to the intermediate-to-late induction of apoptotic genes (4 to 8 hrs) post-irradiation. Case (ii) considers the alternative mechanism whereby late-responsive genes require p53 to recruit specific transcriptional co-factors for their induction, as reviewed recently [67]; the time required to recruit and upregulate the specific co-factors all contribute to the delayed expressions of late p53-responsive genes. Case (iii) accounts for the scenario where a slow rate of transcription leads to corresponding slow protein expression kinetics, and case (iv) considers proteins that take a comparatively longer time to attain steady state upon induction of expression because of their slow degradation rates. Simulation results show that oscillatory p53 induces higher level of gene expressions in all four possible causes of slow dynamics of gene expression (Figure S4 of the *Supplemental Data*).

### p53-transcriptional regulation of apoptosis

**Figure 6:**
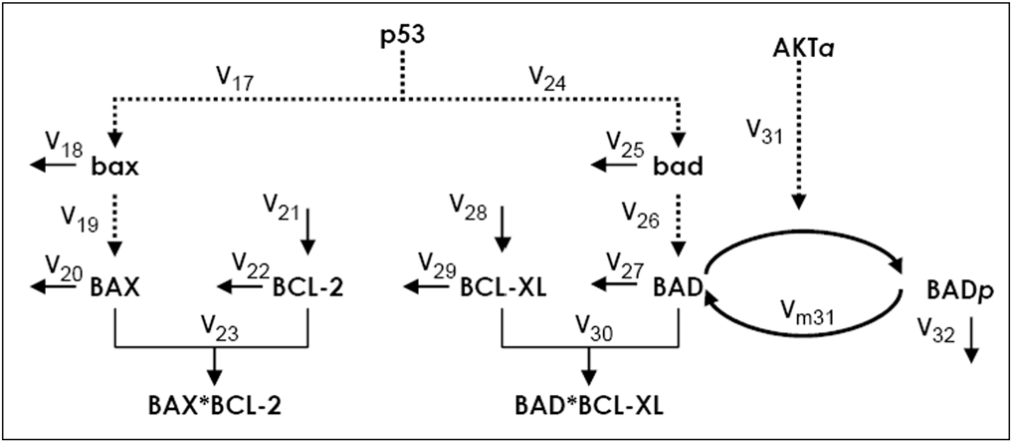
Network model of the regulation of pro-apoptotic genes by p53 and AKT.

Here, we present a model that includes explicitly the downstream signaling pathways from p53 *and* AKT to apoptosis, and study the consequences of p53 oscillations on the regulation of caspase-dependent apoptosis. We focus on the mitochondrial pathway because excessive DNA damage induces apoptosis via the mitochondrial pathway. The role of the BCL2 protein family in regulating this intrinsic pathway is well established. Some members of this family are anti-apoptotic while others are referred to as pro-apoptotic because they bind and inhibit the action of the anti-apoptotic members. DNA damage increases the rate of p53-dependent transcription of pro-apoptotic members such as BAX [31] and BAD [34]. Examples of anti-apoptotic members are BCL-2 [68] and BCL-XL [40], [69]. Excessive downregulation of BCL-2 and BCL-XL triggers caspase-dependent apoptosis [69], [70]. Furthermore, the ratio of the levels of BAX to BCL-2 can be a determinant of apoptosis, as has been illustrated in both experimental and modeling studies [71], [72]. AKT enters the picture because it phosphorylates BAD, thereby inhibiting the binding of BAD with BCL-XL [35], [36], and consequently promoting cell survival. The p53-AKT *Model* is modified to include the aforementioned steps as shown in Figure 6 and Table 2 (the resulting network will be referred to as the *Apoptotic Model*). The following assumptions are made: (1) p53-dependent transcription of bax and bad is assumed to decay exponentially with the same timescale λ as the rate of DNA damage repair. This is because phosphorylation of p53, especially at Serine 46 by DNA damage signal transducers such as ATM and DNA-PK, is needed to express pro-apoptotic proteins (see Figure S1 of the *Supplemental Data*); (2) As p53 first induces the expression of cell-cycle arrest and DNA damage repair genes, followed by pro-apoptotic genes (see [66], [67], [82], [83]), a Hill-type function of time after irradiation is included as a factor in both *v_17_* and *v_24_*. This factor incorporates a time delay for the expressions of bax and bad, following the expressions of mdm2 and pten; (3) The rate equations and kinetic parameters values associated with the reactions of bax, BAX and BCL-2 are set to be identical to those of bad, BAD and BCL-XL, respectively. Note that Table S1 (in *Supplemental Data*) further summarizes and compares the kinetic parameter values used in the *Model* (Figure 1) and the *Apoptotic Model* (Figure 6).

**Table 2:** Kinetic rate equations and parameters of the model depicted in Figure 6.

Differential Equations			Kinetic Parameters
*d[bax]/dt*	* = *	*v_17_ - v_18_*	*k_17_, k_24_ = 0.006*
*d[BAX]/dt*	* = *	*v_19_ - v_20_ - v_23_*	*j_17_, j_24_ = 2*
*d[BCL-2]/dt*	* = *	*v_21_ - v_22_ - v_23_*	*km_17_, km_24_ = 400*
*d[bad]/dt*	* = *	*v_24_ - v_25_*	*k_19_, k_26_ = 0.04*
*d[BAD]/dt*	* = *	*v_26_ - v_27_ - v_30_ - v_31_+v_m31_*	*k_21_, k_28_ = 0.00048*
*d[BADp]/dt*	* = *	*v_31_ - v_m31_ - v_32_*	*k_23_, k_30_ = 600*
*d[BCL-XL]/dt*	* = *	*v_28_ - v_29_ - v_30_*	*k_31_ = 44*
			*j_31_ = 0.01*
*v_17_*	* = *	{k_17_ * [p53]^n3^/(j_17_^n3^+[p53]^n3^)} * {t^ n3^/(km_17_^n3^+t^n3^)} *exp(-λ*t)	*k_m31_ = 0.01*
*v_18_*	* = *	d_bax_ * [bax]	*j_m31_ = 10*
*v_19_*	* = *	k_19_ * [bax]	*d_bax_, d_bad_ = 0.001*
*v_20_*	* = *	d_BAX_ * [BAX]	*d_BAX_, d_BAD_, d_BADp_ = 0.00054*
*v_21_*	* = *	*k_21_*	*d_BCL-2_, d_BCL-XL_ = 0.0036*
*v_22_*	* = *	d_BCL-2_ * [BCL-2]	*n_3_, n_4_ = 4*
*v_23_*	* = *	k_23_ * [BAX] * [BCL-2]	
*v_24_*	* = *	{k_24_ * [p53]^n4^/(j_24_^n4^+[p53]^n4^)} * {t^ n4^/(km_24_^n4^+t^n4^)} *exp(-λ*t)	
*v_25_*	* = *	d_bad_ * [bad]	
*v_26_*	* = *	k_26_ * [bad]	
*v_27_*	* = *	d_BAD_ * [BAD]	
*v_28_*	* = *	*k_28_*	
*v_29_*	* = *	d_BCL-XL_ * [BCL-XL]	
*v_30_*	* = *	k_30_ * [BAD] * [BCL-XL]	
*v_31_*	* = *	k_31_ * [AKTa] * [BAD]/(j_31_+[BAD])	
*v_m31_*	* = *	k_m31_ * [BADp]/(j_m31_+[BADp])	
*v_32_*	* = *	d_BADp_ * [BADp]	

The values of the kinetic parameters associated with the reactions of bax, BAX and BCL-2 are assumed to be identical to those of bad, BAD and BCL-XL, respectively. Time is in *minutes*; concentration is in *µM*.

In the computer simulations, the presence of DNA damage is reflected in the values of the parameters *k_0_* and *d_MDM2a_*. Furthermore, DNA damage is assumed to decay exponentially with timescale λ - this assumption is based on the observation that DNA damage repair reduces the number of DSBs exponentially after exposure to IR doses ranging from 0.2 to 80 Gy [73]. Therefore, both *k_0_* and *d_MDM2a_* are assumed to decay exponentially to their respective basal levels according to the following expressions: k_0_ = k_0,basal_+k_0,IR_*ρ*exp(-λt) and d_MDM2a_ = d_MDM2a,basal_+d_MDM2a,IR_*ρ*exp(-λt).

**Figure 7:**
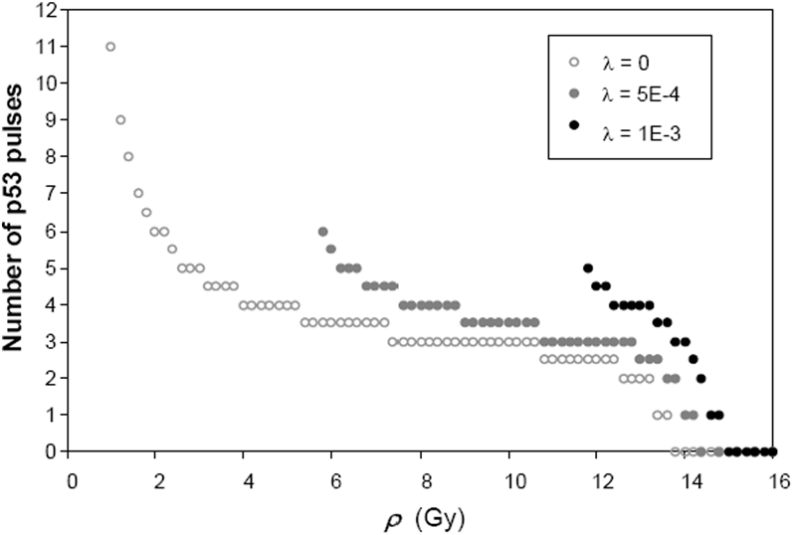
Number of p53 pulses (rounded up to the nearest half pulse, vertical axis) required to deplete BCL-2 below the defined apoptotic threshold level (see text) as function of IR intensity, *ρ*, at various DNA damage repair rates *λ*. Note that the number of p53 pulses becomes zero after the early-switching point.

Representative values of λ are used in the simulations: λ = 0, 0.0005, and 0.001 (corresponding, respectively, to no repair, slow, and moderate repair rates – as inferred from experiments [73]). For all simulations, the initial state of the *Apoptotic Model* is set to its steady state at *ρ* = 0 Gy. In accordance with experimental observations [69]–[72], protein levels of the anti-apoptotic BCL-2 and BCL-XL are used as markers for the progress of apoptosis. For the purpose of exploring the qualitative behavior of the *Apoptotic Model*, and since there are no reported quantitative measurements, we arbitrarily assumed that a cell commits to apoptosis upon the decrease of either BCL-2 or BCL-XL to less than the chosen apoptotic threshold of 1% of its steady state level at *ρ* = 0 Gy. The number of p53 pulses needed to deplete BCL-2 below the apoptotic threshold level is determined. As shown in Figure 7, fewer p53 pulses are required as *ρ* is increased, and in range of *ρ* ∼8 Gy to ∼13 Gy the number of p53 pulses needed are more or less constant. The sharp decrease in the number of p53 pulses for *ρ* >14 Gy is due to the switch to the upper branch of p53 steady states. Interestingly, for fixed *ρ*, more p53 pulses are needed as the repair rate is increased.

As shown in Figure 7, a wide range of *ρ* generates 3 to 4 pulses of p53. To investigate whether this small number of pulses is sufficient to induce higher levels of target gene expression, protein levels of a p53 target gene (X_p_) induced by oscillatory and non-oscillatory p53 are compared at 1200 min (this time period corresponds to 3 to 4 p53 pulses). Indeed, our simulations show that 3 to 4 pulses of p53 are sufficient to induce higher levels of expression of both early and late responsive target genes for the entire range of *ρ* where limit cycles are manifested (see Figure S5 of the *Supplemental Data*). These results hold for all time points.

## Discussion

Our earlier modeling work [41] demonstrated that the p53-AKT network (Figure 1A) has the potential to exhibit switching behavior between pro-survival (high AKT, low p53) and pro-death (high p53, low AKT) states. Originating from the mutual antagonism between p53 and AKT, the switching dynamics is associated with transitions between two stable steady states (bistability) that coexist under the same parameters [41]. Within the p53-AKT network is a negative feedback loop between p53 and MDM2 (steps 5 and 6 in Figure 1A). Kinetic models focusing on this p53-MDM2 loop have been proposed [1], [3], [6]–[9] to explain the experimentally observed oscillations of p53 and MDM2 [1]–[6]; however, the physiological importance of these oscillations remains unclear. The present work is based on the hypothesis that the physiological significance of these p53 oscillations could be found in their role in regulating the switching behavior of the p53-AKT network between pro-survival and pro-death states.

We have shown in this paper that the p53-AKT network (the *Model* shown in Figure 1B), in addition to its potential for bistable behavior, is capable of generating sustained oscillations in p53 and MDM2, and that these oscillations are predicted to occur only within a range of IR intensities (*ρ*) and only in the low-p53 (pro-survival) branch of steady states (Figure 2). Using model parameters that are biologically plausible, the *Model* predicts oscillation periods of 3.5 to 5.2 hrs – well within the reported range of experimental values [3]. We also found that the oscillation frequencies generally increase with increasing *ρ* (Figure S2 of *Supplemental Data*), in accord with experimental observations [3]. In addition, the *Model* reproduces the experimentally measured time separation (or delay) between peaks of p53 and MDM2 oscillations (1.2–1.9 hrs), and, furthermore, predicts that these time delays are insensitive to *ρ* (Figure S2 of the *Supplemental Data*).

Zhang, Brazhnik and Tyson (2007) [8] have suggested models in which the p53-MDM2 negative feedback loop is coupled with a positive loop; in particular, one of these models exhibits multiple steady states as well as large-amplitude sustained oscillations around the high-p53 steady states outside the multiple steady-state regime. These oscillations emerge out of a homoclinic bifurcation [8]. In contrast, our p53-AKT model exhibits sustained oscillations around the low-p53 steady state in both single-steady state and multiple-steady state ranges of parameters (Figure 2); furthermore, these oscillations arise out of supercritical Hopf bifurcation. Two key experimental observations [3] support the Hopf bifurcation scenario: first, observed p53 oscillations show more variability in amplitudes than in periods, and, second, the initial p53 pulse is larger than subsequent pulses, indicating that the initial response to DNA damage is not sluggish (which would be the case if it were a homoclinic bifurcation [74]). Simulations using our model do show that the initial response to DNA damage agrees with the second experimental observation (see also Figure S6 of the *Supplemental Data*); the *Model* generates p53 pulses with larger amplitudes, longer periods and larger time delays between peaks of MDM2 and p53 compared to those generated by a stand-alone p53-MDM2 oscillator (Figure S7 of the *Supplemental Data*). In addition, the *Model* coupled with downstream pathways to apoptosis predict that there is a range of IR intensities where the number of p53 pulses acts as the digital count for the induction of apoptosis (Figure 7).

**Figure 8:**
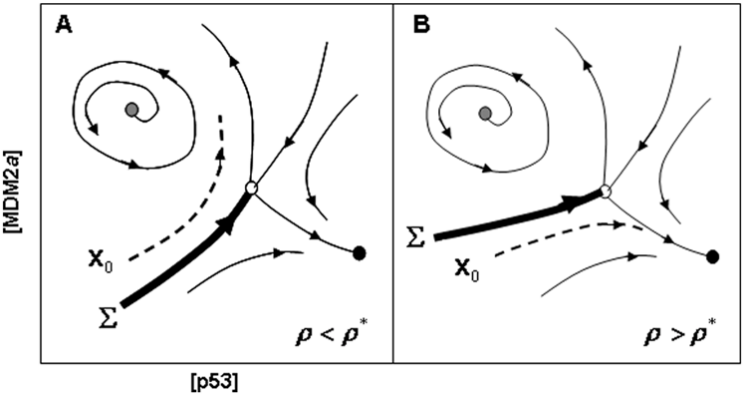
A cartoon of the p53-MDM2*a* phase portrait for the cases *ρ*<ρ* and *ρ*>ρ*, where ρ* is the early-switching point. (A) For *ρ*<ρ*, the initial state corresponding to *ρ* = 0 Gy (indicated by x_0_) is located within the basin of attraction of the limit cycle. (B) At *ρ*>ρ*, x_0_ is now located in the basin of attraction of the high-p53 stable node (•). Note: the actual phase portrait is high-dimensional; the two-dimensional portraits above are given merely to illustrate the basic idea of the origin of the early-switching point.

A subtle prediction of the *Model* comes from the computer-simulated experiments of exposing cells to increasing radiation intensity – subtle because it is not readily evident from the steady state bifurcation diagrams (Figures 2 and 3). In these simulations, the initial condition of the system before IR exposure is always identical to that of the case where *ρ* = 0 (let this initial condition be symbolized by ***x*** *_0_* which is a vector in 8-dimensional concentration space). For values of the parameter *ρ* in between those corresponding to the knees of the steady-state curve in Figure 3, the unstable middle steady state acts as a boundary between the top steady state and oscillatory state. Because the system is multi-dimensional, the boundary that separates the basins of attractions of the top steady state and oscillatory state (for *ρ*<16, see Figure 3) is a multidimensional surface – let us call this surface ***Σ*** . The position of ***Σ*** depends on *ρ*, and as *ρ* is increased, the position of ***x*** *_0_* relative to ***Σ*** moves from one that is located in the basin of attraction of the oscillatory state to one on the other side of ***Σ*** that is within the basin of attraction of the top steady state; this switch occurs at ρ*, the *early-switching point* (so-called because ρ* is less than the *ρ* corresponding to the right knee of the steady state curve). A cartoon that illustrates the aforementioned ideas is shown in Figure 8. A scattering of initial conditions around ***x*** *_0_* leads to various probabilities of switching from the oscillatory state to the top steady state; for example, as shown in Figure 4, about half of all sampled initial conditions around ***x*** *_0_* are attracted to the top steady state at *ρ* = ρ* = 13.8 Gy (Figure 4) which is significantly lower than the value of 20.8 Gy corresponding to the right-knee of the steady-state curve (Figure 2). These simulations suggest that because of the instability of the lower steady states where oscillations exist (Figures 2A and 3), the fluctuations in p53 and MDM2 increase the probability of crossing ***Σ*** – causing the abovementioned *early switch* which can be interpreted as a decrease in tolerance to death-inducing IR.

Another important result presented in this paper is the consequence of p53 oscillations on the level of expression of p53-target genes. It is predicted that at a fixed *ρ* where oscillations exist, p53-target genes are expressed at higher levels compared to the case where there are no oscillations (Figure 5B). Furthermore, this increase in expression of target genes is more pronounced as the amplitude of p53 oscillations becomes larger (Figure 5C). In the context of the p53-AKT network (Figure 1A), the model predicts an increase in PTEN expression (thereby promoting the antagonism against the survival factor AKT) and an increase in BAX and BAD expression (thereby promoting apoptosis). Thus, IR exposure that induces p53 oscillations is predicted to sensitize cells towards apoptotic death. The issue of how p53 oscillations may influence the determination of cell fate (i.e., between cell cycle arrest/DNA damage repair versus apoptosis) is not considered in the present work.

Reported differential sensitivity of mice organs to IR-induced apoptosis [75] suggests that IR sensitivity is closely linked with p53 oscillations and the proliferation status of cells. The early-switch phenomenon and the increased expression of p53-target genes due to p53 oscillations, as discussed in the preceding paragraph, may be offered as an explanation for this IR-sensitivity. In terms of the proliferation status of cells, highly IR-sensitive organs such as spleen and small intestine – in which p53 oscillations have been observed [4] – are generally composed of proliferating cells, whereas non-proliferating cells in organs such as the brain are generally IR-insensitive. Moreover, *in vitro* studies of p53 oscillations have so far been carried out using proliferating cells such as breast cancer epithelial [1], [3], [5], [6] and fibroblast cells [2]. The fact that our *Model* predicts that the oscillations occur around the low-p53 steady states is consistent with these observed association between proliferating cells and p53 oscillations.

We would like to offer ways to test the predictions of our model in the laboratory. Several single cell time-lapse microscopy experiments (e.g., Ref. [3]) could be performed to validate key hypotheses generated from the models by taking advantage of the evidence that not every cell (of the same type) would manifest p53-MDM2 oscillations when irradiated with the same IR intensity. For instance, only 40% of MCF-7 cells showed oscillations upon 10 Gy of gamma radiation [3]. Strictly speaking, a cell could only be in one of the three states: low-p53 (non-oscillatory cells), high-p53 and oscillatory p53 (oscillatory cells). The key prediction that p53 oscillations induce higher level of target gene expression could be tested by semi-quantifying the expression level of a (transfected) luciferase reporter gene that possesses a p53 promoter sequence upon irradiation. Oscillatory cells are predicted to express higher intensity of fluorescence than non-oscillatory cells (of the same type); comparisons should be made among cells that expressed similar mean level of p53. Also, an interesting experiment would be to test whether higher p53-dependent expression of cell cycle and DNA repair genes in oscillatory cells could lead to faster cell cycle arrest and repair damage DNA than non-oscillatory cells upon irradiation. In contrast, the other key prediction that p53 oscillations lower the IR intensity level at which the system switches to high-p53 state is relatively trickier to perform experimentally. It involves the determination of cumulative IR dose that lead to a high-p53 state in each oscillatory and non-oscillatory cells by increasing the IR dose gradually. Oscillatory cells are predicted to switch to high-p53 state over a wider range of cumulative IR dose with lower median than non-oscillatory cells.

Lastly, for the future development of our model, we would like to point out that besides PTEN, the insulin growth factor-binding protein 3 (IGFBP3) connects p53 to AKT. Upon DNA damage, IGFBP3 is upregulated by both p53-dependent and independent transcription [76], [77]. IGFBP3 binds and sequesters IGFs (insulin growth factors) away from IGFRs (insulin growth factor receptors), and thereby inhibits AKT activation; active IGF-bound IGFRs induce the downstream activation of the PI3K/AKT survival pathway [78]. Surprisingly, through unknown mechanisms, IGFBP3 could also sensitize cells to the phosphorylation of AKT by IGFs, which leads to AKT activation [79], [80]. Thus, further experimental studies are needed to resolve the conflicting relationship between p53, IGFBP3 and AKT.

## Materials and Methods

To determine the steady states of the *Model*, the left-hand sides of the ODEs in Table 1 are all set to zero and the corresponding systems of nonlinear algebraic equations were solved numerically using Maple (version 7.0). The steady states as functions of certain parameters are referred to as steady-state bifurcation diagrams. The local stability of the steady states is determined using standard linear stability analysis, involving the determination of the eigenvalues of associated Jacobian matrices. To obtain the time-series trajectories of the species concentrations in the model, the ODEs are integrated using a modified Rosenbrock formula of order 2 that is implemented in the MATLAB (The MathWorks, Natick, MA) platform (version 6.5, Release 13).

## Supporting Information

Table S1 (0.13 MB DOC)

Figure S1 (0.05 MB DOC)

Figure S2 (0.03 MB DOC)

Figure S3 (0.09 MB DOC)

Figure S4 (0.08 MB DOC)

Figure S5 (0.08 MB DOC)

Figure S6 (0.08 MB DOC)

Figure S7 (0.04 MB DOC)
