# Supplementary material for: Oscillations of the p53-Akt Network: Implications on Cell Survival and Death
Source: PLoS One. 2009 Feb 6;4(2):e4407. doi: 10.1371/journal.pone.0004407 (PMC2634840; doi:10.1371/journal.pone.0004407)
Supplement: Table S1 — (0.13 MB DOC) [file pone.0004407.s001.doc]

**Table S1**. The 59 parameters used in the model simulations for the *Model* and the *Apoptotic Model*.

Par (column 2) denotes kinetic parameter. Items 1 to 34 are used in the *Model* while items 35 onwards are used in the *Apoptotic Model*. In Range (column 6), values in parentheses indicate the range of kinetic parameter values varied in Ref. S58 for conservation of bistability; parameters whose values were obtained directly from experiments were not varied. Note: References cited in this table are listed immediately after this table.

| **Item** | **Par** | **Description** | **Units** | **Value Used** | **Range** | **Refs** | **Note** |
| --- | --- | --- | --- | --- | --- | --- | --- |
| **1** | *k_0,basal_* | Production of active p53 under no DNA damage | µM/min | 0.02 | 0.005 - 0.2  (0.08 - 0.12) | S43 | 0.1 in Ref. S58 |
| **2** | *k_1_* | Inactivation of active p53 (degradation or dephosphorylation) | /min | 0.02 | 0.02 – 0.2, 0.05 | S43, S51, S53 (Direct measurements) | 0.05 in Ref. S58 |
| **3** | *k_2_* | MDM2*a*-dependent degradation of p53 | /min | 0.065 | 0.0184 - 0.092  (0.044 - 0.066) | S43 | 0.055 in Ref. S58 |
| **4** | *j_2_* | Michaelis constant of MDM2-dependent degradation of p53 | µM | 0.01 | 0.03 - 0.3  (0.08 - 0.12) | S43 | 0.1 in Ref. S58 |
| **5** | *k_3_* | Degradation of MDM2 | /min | 0.005 | 0.0028, 0.0347  (0.012 - 0.018) | S43, S53 | 0.015 in Ref. S58 |
| **6** | *k_4_* | Basal induction of mdm2 | µM/min | 0.0009 | 0.0009 | S43 | New |
| **7** | *k_5_* | p53-dependent transcription of mdm2 | µM/min | 0.0375 | 0.024  (0.0192 - 0.0288) | S43 | 0.024 in Ref. S58 |
| **8** | *j_5_* | Dissociation constant of p53-dependent transcription of mdm2 | µM | 0.5 | ~1  (0.8 – 1.2) | S43 | 1 in Ref. S58 |
| **9** | *k_6_* | AKT*a* phosphorylation of MDM2 | /min | 22 | 22 | S55 (Direct measurement) | 10 in Ref. S58 |
| **10** | *j_6_* | Michaelis constant of AKT*a* phosphorylation of MDM2 | µM | 0.6 | 0.6 | S55 (Direct measurement) | 0.3 in Ref. S58 |
| **11** | *k_m6_* | Dephosphorylation of MDM2*a* | µM/min | 0.5 | 0.0000297 - 2.92  (0.16 - 0.24) | S44, S47 | 0.2 in Ref. S58 |
| **12** | *j_m6_* | Michaelis constant of dephosphorylation of MDM2*a* | µM | 0.1 | 0.00238 - 2.23  (0.08 - 0.12) | S44 – S47, S48 | As in Ref. S58 |
| **13** | *k_7_* | Degradation of mdm2 | /min | 0.01 | 0.01 | S43 | New |
| **14** | *k_8_* | PIP3-mediated phosphorylation of AKT | /min | 20 | 20  (16 - 24) | S48 | As in Ref. S57 |
| **15** | *j_8_* | Michaelis constant of PIP3-mediated phosphorylation of AKT | µM | 0.1 | 0.1  (0.08 - 0.12) | S48 | As in Ref. S58 |
| **16** | *k_m8_* | Dephosphorylation of AKT*a* | µM/min | 0.2 | 0.0000297 - 2.92  (0.16 - 0.24) | S44, S47 | As in Ref. S58 |
| **17** | *j_m8_* | Michaelis constant of dephosphorylation of AKT*a* | µM | 0.1 | 0.1  (0.08 - 0.12) | S48 | As in Ref. S58 |
| **18** | *k_9,basal_* | Degradation of MDM2*a* under no DNA damage | /min | 0.005 | 0.0028, 0.0347  (0.012 - 0.018) | S43, S53 | 0.015 in Ref. S58 |
| **19** | *k_10_* | Translation of mdm2 to MDM2 | /min | 0.02 | 0.02 | S43 | New |
| **20** | *k_11_* | p53-dependent transcription of pten | µM/min | 0.006 | 0.006 | S50 (Direct measurement) | As in Ref. S58 |
| **21** | *j_11_* | Dissociation constant of p53-dependent transcription of pten | µM | 2 | > 1  (1.6 - 2.4) | S50 | As in Ref. S58 |
| **22** | *k_12_* | Basal induction of pten | µM/min | 0.0009 | 0.0009 | Assumed to be identical to *k_4_* | New |
| **23** | *k_13_* | Degradation of pten | /min | 0.01 | 0.01 | Assumed to be identical to *k_7_* | New |
| **24** | *k_14_* | Translation of pten to PTEN | /min | 0.02 | 0.02 | Assumed to be identical to *k_10_* | New |
| **25** | *k_15_* | Degradation of PTEN | /min | 0.0054 | 0.0025 -0.0083 | S52 (Direct measurement) | As in Ref. S58 |
| **26** | *k_16_* | Phosphorylation of PIP2 | µM/min | 0.15 | 0.15  (0.12 - 0.18) | S47 | As in Ref. S58 |
| **27** | *j_16_* | Michaelis constant of phosphorylation of PIP2 | µM | 0.1 | 0.1  (0.08 - 0.12) | S48 | As in Ref. S58 |
| **28** | *k_m16_* | PTEN dephosphorylation of PIP3 | /min | 73 | 42.1, 73 ± 4.4 | S48, S49 (Direct measurement) | As in Ref. S58 |
| **29** | *j_m16_* | Michaelis constant of PTEN dephosphorylation of PIP3 | µM | 0.5 | 0.1 - 1  (0.4 - 0.6) | S48, S52, S54 | As in Ref. S58 |
| **30** | *n1* | Hill coefficient of p53-dependent transcription of mdm2 | - | 4 | 3, 4 | S43 | 3 in Ref. S58 |
| **31** | *n2* | Hill coefficient of p53-dependent transcription of pten | - | 4 | 3, 4 | S43 | 3 in Ref. S58 |
| **32** | [PIP*_T_*] | Sum of [PIP2] and [PIP3] | µM | 1 | (0.5 - 2) | Arbitrary | As in Ref. S58 |
| **33** | *k_0,IR_* | Proportional constant between IR intensity (*ρ*) and *k_0_* | µM  /min  /Gy | 0.0025 |  | Arbitrary | New |
| **34** | *k_9,IR_* | Proportional constant between IR intensity (*ρ*) and *k_9_* | /min  /Gy | 0.0025 |  | Arbitrary | New |
| **35** | *k_17_* | p53-dependent transcription of bax | µM/min | 0.006 | ~ 0.006 | Assumed same order of magnitude as *k_11_* | New |
| **36** | *j_17_* | Dissociation constant of p53-dependent transcription of bax | µM | 2 | > 1 | Assumed same order of magnitude as *j_11_* | New |
| **37** | *k_m17_* | Time-delay in p53-dependent transcription of bax | min | 400 | ~ one p53 pulse | S56 | New |
| **38** | *k_18_* | Degradation of bax | /min | 0.001 | ~ 0.001 | Assumed 1 order of magnitude smaller than *k_7_* and *k_13_* | New |
| **39** | *k_19_* | Translation of bax to BAX | /min | 0.04 | ~ 0.02 | Assumed same order of magnitude as *k_10_* and *k_14_* | New |
| **40** | *k_20_* | Degradation of BAX | /min | 0.00054 | ~ 0.0005 | Assumed 1 order of magnitude smaller than *k_15_*, *k_3_* and *k_9_* | New |
| **41** | *k_21_* | Basal induction of BCL-2 | µM/min | 0.00048 | ~ 0.0001 | Assumed 1 order of magnitude smaller than *k_8_* | New |
| **42** | *k_22_* | Degradation of BCL-2 | /min | 0.0036 | ~ 0.005 | Assumed same order of magnitude as *k_15_*, *k_3_* and *k_9_* | New |
| **43** | *k_23_* | Formation of BAX*BCL-2 complex | / (µMmin) | 600 | 600 | S57 | New |
| **44** | *k_24_* | p53-dependent transcription of bad | µM/min | 0.066 |  | = *k_17_* | New |
| **45** | *j_24_* | Dissociation constant of p53-dependent transcription of bad | µM | 2 |  | = *j_17_* | New |
| **46** | *k_m24_* | Time-delay in p53-dependent transcription of bad | min | 400 |  | = *k_m17_* | New |
| **47** | *k_25_* | Degradation of bad | /min | 0.001 |  | = *k_18_* | New |
| **48** | *k_26_* | Translation of bad to BAD | /min | 0.04 |  | = *k_19_* | New |
| **49** | *k_27_* | Degradation of BAD | /min | 0.00054 |  | = *k_20_* | New |
| **50** | *k_28_* | Basal induction of BCL-XL | µM/min | 0.00048 |  | = *k_21_* | New |
| **51** | *k_29_* | Degradation of BCL-XL | /min | 0.0036 |  | = *k_22_* | New |
| **52** | *k_30_* | Formation of BAD*BCL-XL complex | / (µMmin) | 600 |  | = *k_23_* | New |
| **53** | *k_31_* | AKT*a* phosphorylation of BAD | /min | 44 | 44, 0.42 - 64.8 | Assumed same order of magnitude as *k_6_* | New |
| **54** | *j_31_* | Michaelis constant of AKT*a* phosphorylation of BAD | µM | 0.01 | 0.06, 0.00357 - 146 | Assumed 1 order of magnitude smaller than *j_6_* | New |
| **55** | *k_m31_* | Dephosphorylation of BAD*p* | µM/min | 0.01 | ~ 0.01, 0.0000297 - 2.92 | Assumed 1 order of magnitude smaller than *k_m6_* and *k_m8_* | New |
| **56** | *j_m31_* | Michaelis constant of dephosphorylation of BAD*p* | µM | 10 | ~ 10 | Assumed 3 orders of magnitude larger than *j_m6_* and *j_m8_* | New |
| **57** | *k_32_* | Degradation of BAD*p* | /min | 0.00054 |  | = *k_27_* | New |
| **58** | *n3* | Hill coefficient of p53-dependent transcription of bax | - | 4 |  | = *n1, n2* | New |
| **59** | *n4* | Hill coefficient of p53-dependent transcription of bad | - | 4 |  | = *n3* | New |

**References Cited in Table S1.**

S43. Ma L, Wagner J, Rice JJ, Hu W, Levine AJ, Stolovitzky GA (2005) A plausible model for the digital response of p53 to DNA damage. Proc Natl Acad Sci USA 102: 14266–14271.

S44. Qiu D, Mao L, Kikuchi S, Tomita M (2004) Sustained MAPK activation is dependent on continual NGF receptor regeneration. Dev Growth Differ 46: 393–403.

S45. Schoeber B, Eichler-Jonsson C, Gilles ED, Muller G (2002) Computational modeling of the dynamics of the MAP kinase cascade activated by surface and internalized EGF receptors. Nat Biotechnol 20: 370–375.

S46. Markevich NI, Hoek JB, Kholodenko BN (2005) Signaling switches and bistability arising from multisite phosphorylation in protein kinase cascades. J Cell Biol 164: 353–359.

S47. Kholodenko BN (2000) Negative feedback and ultrasensitivity can bring about oscillations in the mitogen-activated protein kinase cascades. Eur J Biochem 267: 1583–1588.

S48. Giri L, Mutalik VK, Venkatesh KV (2004) A steady state analysis indicates that negative feedback regulation of PTP1B by Akt elicits bistability in insulinstimulated GLUT4 translocation. Theor Biol Med Model 1: 2.

S49. McConnachie GIP, Walker SM, Downes CP (2003) Interfacial kinetic analysis of the tumour suppressor phosphatase, PTEN: evidence for activation by anionic phospholipids. Biochem J 371: 947–955.

S50. Stambolic V, MacPherson D, Sas D, Lin Y, Snow B, Jang Y, Benchimol S, Mak TW (2001) Regulation of PTEN Transcription by p53. Mol Cell 8: 317–325.

S51. Zhou BP, Liao Y, Xia W, Zou Y, Spohn B, Hung MC (2001) HER-2/neu induces p53 ubiquitination via Akt-mediated MDM2 phosphorylation. Nat Cell Biol 3: 973–982.

S52. Georgescu MM, Kirsch KH, Akagi T, Shishido T, Hanafusa H (1999) The tumor-suppressor activity of PTEN is regulated by its carboxyl-terminal region. Proc Natl Acad Sci USA 96: 10182–10187.

S53. Bar-Or RL, Maya R, Segel LA, Alon U, Levine AJ, Oren M (2000) Generation of oscillations by the p53-Mdm2 feedback loop: A theoretical and experimental study. Proc Natl Acad Sci U S A 97: 11250–11255.

S54. Vazquez F, Ramaswamy S, Nakamura N, Sellers WR (2000) Phosphorylation of the PTEN tail regulates protein stability and function. Mol Cell Biol 20: 5010–5018.

S55. Zhang X, Zhang S, Yamane H, Wahl R, Ali A, Lofgren JA, Kendall RL (2006) Kinetic Mechanism of AKT/PKB Enzyme Family. J Biol Chem 281: 13949–13956.

S56. Zhao R, Gish K, Murphy M, Yin Y, Notterman D, Hoffman WH, Tom E, Mack DH, Levine AJ (2000) Analysis of p53-regulated gene expression patterns using oligonucleotide arrays. Genes Dev 14: 981–993.

S57. Bagci EZ, Vodovotz Y, Billiar TR, Ermentrout GB, Bahar I (2006) Bistability in Apoptosis - Roles of Bax, Bcl-2, and Mitochondrial Permeability Transition Pores. Biophys J 90: 1546–1559.

S58. Wee KB, Aguda BD (2006) Akt versus p53 in a network of oncogenes and tumor suppressor genes regulating cell survival and death. Biophys J 91: 857–865.
