# Supplementary material for: Oscillations of the p53-Akt Network: Implications on Cell Survival and Death
Source: PLoS One. 2009 Feb 6;4(2):e4407. doi: 10.1371/journal.pone.0004407 (PMC2634840; doi:10.1371/journal.pone.0004407)
Supplement: Figure S1 — (0.05 MB DOC) [file pone.0004407.s002.doc]

***DNA damage signal transduction pathways***

Upon IR, the major cellular events occurs generally in the following sequence: formation and detection of DSBs, transmission of DNA damage signal by transduction pathways and finally, cellular outcomes such as cell cycle arrest, damage repair and apoptosis executed by downstream effectors [S1] – [S3]. The biochemical and biophysical mechanisms of these events are however far from being completely elucidated [S2], [S4], [S5]. Despite that, existing notion of the DNA damage signal transduction pathways regulating post-translation modifications of p53 and MDM2, which is of most relevance in this study, is complex (Figure S1). In general, the part lists of the damage signal transduction pathway can be categorized into primary and secondary transducers.

**ATR**

**ATM**

**p53**

**MDM2**

**CHK2**

**DNA-PK**

**BRCA1**

**53BP1**

**PP2A**

**Figure S1**. **DNA damage signal transduction pathways regulating p53 and MDM2.**

Signal transduction pathways regulating the activation (arrows) and suppression (hammerheads) of p53 and MDM2, following IR-induced formation of DSBs. The primary signal transducers are ATM, ATR and DNA-PK. However, crosstalking among the secondary signal transducers are prevalent, as indicated by the numerous network edges.

ATM, ATR and DNA-PK kinases are primary transducers, which belong to the phosphatidylinositol kinase-related kinase family. ATM is present as inactive dimers in undamaged cells but is rapidly phosphorylated and thereby dissociates into active monomers in the presence of DSBs [S6]. Active ATM phosphorylates p53 [S7], [S8], MDM2 [S9], [S10], CHK2 [S11], [S12], BRCA1 [S13], 53BP1 [S14] and maybe AKT [S15], [S16]. p53 is stabilized and activated after being phosphorylated at multiple serine residues [S7] namely at, 9, 15 (inhibits binding to MDM2), 20 and 46 (important for apoptotic activity). On the other hand, ATM-mediated phosphorylation of MDM2 increases the latter auto-ubiquitination [S9], [S10]. Although ATM is a key signal transducer in the DNA damage response, alternate pathways exist as ATM-null cells treated with IR still show phosphorylations of p53, MDM2, BRCA1 and CHK2 albeit with delayed kinetics [S2]. For instance, ATR has been implicated in the late phosphorylation of p53 at Serine15 after IR [S17] – [S19]. Moreover, DNA-PK could bind to DSBs [S2], [S5] after which it phosphorylates MDM2 at Serine17 (negates binding to p53) [S10], [S20] and p53 at serine residues at 15, 37, 46 and 392 [S8], [S21] – [S23]. Indeed, DNA-PK deficient cells display delayed and attenuated p53-dependent activation of p21 and MDM2 proteins [S24].

Secondary signal transducers are direct downstream substrates of primary transducers, which consist of CHK2, 53BP1 and BRCA1 (Figure S1). In particular, CHK2 is a common target of ATM and DNA-PK [S25]. CHK2 exists as inactive monomers in unperturbed cells [S26] but undergoes multiple intermolecular phosphorylations especially at Threonine68 following DSBs formation [S27], [S28], after which it dimerizes and becomes fully activated [S28], [S29]. Active CHK2 phosphorylates p53 at Serine20 and thereby blocks p53-MDM2 interaction [S30] – [S34]; moreover, by cooperating with DNA-PK, CHK2 could also phosphorylate p53 at Serine15 [S8], [S22]. Similarly, BRCA1 phosphorylates p53 efficiently at Serine15 after activation by ATM [S35], [S36]. 53BP1, on the other hand, is required for p53 accumulation in response to IR [S37]. Remarkably, crosstalks among the secondary signal transducers exist. BRCA1 is phosphorylated by CHK2 [S38] while 53BP1 played a partially redundant role in phosphorylating BRCA1 and CHK2 [S37].

Opposing the effects of kinases are de-phosphorylating enzymes called phosphatases such as PP2A and WIP1 that extinguish the DNA damage signals by deactivating CHK2 and p53 (Figure S1). PP2A dephosphorylates CHK2 at Threonine68 [S39], which prevents the activation of inactive CHK2, but does not abolish kinase activity of active CHK2. In addition, active CHK2 is dephosphorylated by WIP1 at Threonine68, Serine516 and Serine33 [S40]. Interestingly, WIP1 is involved in a negative feedback loop with p53, i.e., p53 activates the transcription of WIP1 [S41] whereas WIP1 dephosphorylates p53 at Serine15 [S42]. Clearly, there are redundancies in the DNA damage signal transduction pathways. Nevertheless, not all of the pathways may be simultaneously active and their relative contributions in the post-translation modifications of p53 and MDM2 proteins could depend on cell lines and extent of DNA damage.

**References**:

1. Norbury CJ, Zhivotovsky B (2004) DNA damage-induced apoptosis. Oncogene 23: 2797–2808.
2. Zhou BS, Elledge SJ (2000) The DNA damage response: putting checkpoints in perspective. Nature 408: 433–439.
3. Rouse J, Jackson SP (2002) Interfaces Between the Detection, Signaling, and Repair of DNA Damage. Science 297: 547–551.
4. McGowan CH, Russell P (2004) The DNA damage response: sensing and signaling. Curr Opin Cell Biol 16:629–633
5. Durocher D, Jackson SP (2001) DNA-PK, ATM and ATR as sensors of DNA damage: variations on a theme? Curr Opin Cell Biol 13: 225–231.
6. Bakkenist CJ, Kastan MB (2003) DNA damage activates ATM through intermolecular autophosphorylation and dimer dissociation. Nature 421: 499–506.
7. Saito S, Goodarzi AA, Higashimoto Y, Noda Y, Lees-Miller SP, Appella E, Anderson CW (2002) ATM mediates phosphorylation at multiple p53 sites, including Ser(46), in response to ionizing radiation. J Biol Chem 277: 12491–12494.
8. Kapoor M, Hamm R, Yan W, Taya Y, Lozano G (2000) Cooperative phosphorylation at multiple sites is required to activate p53 in response to UV radiation. Oncogene 19: 358–364.
9. Khosravi R, Maya R, Gottlieb T, Oren M, Shiloh Y, Shkedy D (1999) Rapid ATM-dependent phosphorylation of MDM2 precedes p53 accumulation in response to DNA damage. Proc Natl Acad Sci U S A 96: 14973–14977.
10. Stommel JM, Wahl GM (2004) Accelerated MDM2 auto-degradation induced by DNA-damage kinases is required for p53 activation. EMBO J 23: 1547–1556.
11. Matsuoka S, Huang M, Elledge SJ (1998) Linkage of ATM to cell cycle regulation by the Chk2 protein kinase. Science 282: 1893–1897.
12. Ahn JY, Schwarz JK, Piwnica-Worms H, Canman CE (2000) Threonine 68 phosphorylation by ataxia telangiectasia mutated is required for efficient activation of Chk2 in response to ionizing radiation. Cancer Res 60: 5934–5936.
13. Cortez D, Wang Y, Qin J, Elledge SJ (1999) Requirement of ATM-dependent phosphorylation of brca1 in the DNA damage response to double-strand breaks. Science 286: 1162–1166.
14. Fernandez-Capetillo O, Chen HT, Celeste A, Ward I, Romanienko PJ, Morales JC, Naka K, Xia Z, Camerini-Otero RD, Motoyama N, Carpenter PB, Bonner WM, Chen J, Nussenzweig A (2002) DNA damage-induced G2-M checkpoint activation by histone H2AX and 53BP1. Nat Cell Biol 4: 993–997.
15. Fayard E, Tintignac LA, Baudry A, Hemmings BA (2006) Protein kinase B/Akt at a glance. J Cell Sci 118: 5675–5678.
16. Viniegra JG, Martinez N, Modirassari P, Losa JH, Parada Cobo C, Lobo VJ, Luquero CI, Alvarez-Vallina L, Ramon y Cajal S, Rojas JM, Sánchez-Prieto R (2005) Full activation of PKB/Akt in response to insulin or ionizing radiation is mediated through ATM. J Biol Chem 280: 4029–4036.
17. Tibbetts RS, Brumbaugh KM, Williams JM, Sarkaria JN, Cliby WA, Shieh SY, Taya Y, Prives C, Abraham RT (1999) A role for ATR in the DNA damage-induced phosphorylation of p53. Genes Dev 13: 152–157.
18. Lakin ND, Hann BC, Jackson SP (1999) The ataxia-telangiectasia related protein ATR mediates DNA-dependent phosphorylation of p53. Oncogene 18: 3989–3995.
19. Hall-Jackson CA, Cross DA, Morrice N, Smythe C (1999) ATR is a caffeine-sensitive, DNA-activated protein kinase with a substrate specificity distinct from DNA-PK. Oncogene 18: 6707–6713.
20. Mayo LD, Turchi JJ, Berberich SJ (1997) Mdm-2 phosphorylation by DNA-dependent protein kinase prevents interaction with p53. Cancer Res 57: 5013–5016.
21. Komiyama S, Taniguchi S, Matsumoto Y, Tsunoda E, Ohto T, Suzuki Y, Yin HL, Tomita M, Enomoto A, Morita A, Suzuki T, Ohtomo K, Hosoi Y, Suzuki N (2004) Potentiality of DNA-dependent protein kinase to phosphorylate Ser46 of human p53. Biochem Biophys Res Commun 323: 816–822.
22. Jack MT, Woo RA, Motoyama N, Takai H, Lee PW (2004) DNA-dependent protein kinase and checkpoint kinase 2 synergistically activate a latent population of p53 upon DNA damage. J Biol Chem 279: 15269–15273.
23. Shieh S-Y, Ikeda M, Taya Y, Prives C (1997) DNA Damage-Induced Phosphorylation of p53 Alleviates Inhibition by MDM2. Cell 91: 325–334.
24. Kachnic LA, Wu B, Wunsch H, Mekeel KL, DeFrank JS, Tang W, Powell SN (1999) The ability of p53 to activate downstream genes p21(WAF1/cip1) and MDM2, and cell cycle arrest following DNA damage is delayed and attenuated in scid cells deficient in the DNA-dependent protein kinase. J Biol Chem 274: 13111–13117.
25. Li J, Stern DF (2005) Regulation of CHK2 by DNA-dependent protein kinase. J Biol Chem 280: 12041–12050.
26. Ahn J, Urist M, Prives C (2004) The Chk2 protein kinase. DNA Repair 3: 1039–1047.
27. Lee CH, Chung JH (2001) The hCds1 (Chk2)-FHA domain is essential for a chain of phosphorylation events on hCds1 that is induced by ionizing radiation. J Biol Chem 276: 30537–30541.
28. Schwarz JK, Lovly CM, Piwnica-Worms H (2003) Regulation of the Chk2 Protein Kinase by Oligomerization-Mediated cis- and trans-Phosphorylation, Mol Cancer Res 1: 598–609.
29. Wu X, Chen J (2003) Autophosphorylation of checkpoint kinase 2 at serine 516 is required for radiation-induced apoptosis, J Biol Chem 278: 36163–36168.
30. Caspari T (2000) How to activate p53. Curr Biol 10: R315–R317.
31. Hirao A, Kong YY, Matsuoka S, Wakeham A, Ruland J, Yoshida H, Liu D, Elledge SJ, Mak TW (2000) DNA damage-induced activation of p53 by the checkpoint kinase Chk2. Science 287: 1824–1827.
32. Chehab NH, Malikzay A, Appel M, Halazonetis TD (2000) Chk2/hCds1 functions as a DNA damage checkpoint in G(1) by stabilizing p53. Genes Dev 14: 278–288.
33. Shieh SY, Ahn J, Tamai K, Taya Y, Prives C (2000) The human homologs of checkpoint kinases Chk1 and Cds1 (Chk2) phosphorylate p53 at multiple DNA damage-inducible sites. Genes Dev 14: 289–300.
34. Takai H, Naka K, Okada Y, Watanabe M, Harada N, Saito S, Anderson CW, Appella E, Nakanishi M, Suzuki H, Nagashima K, Sawa H, Ikeda K, Motoyama N (2002) Chk2-deficient mice exhibit radioresistance and defective p53-mediated transcription. EMBO J 21: 5195–5205.
35. Foray N, Marot D, Gabriel A, Randrianarison V, Carr AM, Perricaudet M, Ashworth A, Jeggo P (2003) A subset of ATM- and ATR-dependent phosphorylation events requires the BRCA1 protein. EMBO J 22: 2860–2871.
36. Fabbro M, Savage K, Hobson K, Deans AJ, Powell SN, McArthur GA, Khanna KK (2004) BRCA1-BARD1 complexes are required for p53Ser-15 phosphorylation and a G1/S arrest following ionizing radiation-induced DNA damage. J Biol Chem 279: 31251–31258.
37. Wang B, Matsuoka S, Carpenter PB, Elledge SJ (2002) 53BP1, a mediator of the DNA damage checkpoint. Science 298: 1435–1438.
38. Lee JS, Collins KM, Brown AL, Lee CH, Chung JH (2000) hCds1-mediated phosphorylation of BRCA1 regulates the DNA damage response. Nature 404: 201–204.
39. Liang X, Reed E, Yu JJ (2006) Protein phosphatase 2A interacts with Chk2 and regulates phosphorylation at Thr-68 after cisplatin treatment of human ovarian cancer cells. Int J Mol Med 17: 703–708.
40. Oliva-Trastoy M, Berthonaud V, Chevalier A, Ducrot C, Marsolier-Kergoat M-C, Mann C, Leteurtre F (2007) The Wip1 phosphatase (PPM1D) antagonizes activation of the Chk2 tumour suppressor kinase. Oncogene 26: 1449–1458.
41. Fiscella M, Zhang H, Fan S, Sakaguchi K, Shen S, Mercer WE, Vande Woude GF, O'Connor PM, Appella E (1997) Wip1, a novel human protein phosphatase that is induced in response to ionizing radiation in a p53-dependent manner. Proc Natl Acad Sci U S A 94: 6048–6053.
42. Lu X, Nannenga B, Donehower LA (2005) PPM1D dephosphorylates Chk1 and p53 and abrogates cell cycle checkpoints. Genes Dev 19: 1162–1174.
