# Supplementary material for: Oscillations of the p53-Akt Network: Implications on Cell Survival and Death
Source: PLoS One. 2009 Feb 6;4(2):e4407. doi: 10.1371/journal.pone.0004407 (PMC2634840; doi:10.1371/journal.pone.0004407)
Supplement: Figure S2 — (0.03 MB DOC) [file pone.0004407.s003.doc]

***ρ* (Gy)**

**Figure S2**. Oscillation properties of p53, mdm2, MDM2 and MDM2*a* are depicted for the entire range of *ρ* where the *Model* exhibits limit cycles. Limit cycle periods of p53, mdm2, MDM2 and MDM2*a* are identical as shown here in gray. The remaining curves depict the time-delays of the peaks of mdm2, MDM2 and MDM2*a* pulses relative to the peaks of p53 pulses (see inset).
