# Supplementary material for: Oscillations of the p53-Akt Network: Implications on Cell Survival and Death
Source: PLoS One. 2009 Feb 6;4(2):e4407. doi: 10.1371/journal.pone.0004407 (PMC2634840; doi:10.1371/journal.pone.0004407)
Supplement: Figure S3 — (0.09 MB DOC) [file pone.0004407.s004.doc]

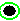

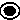

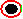
**Figure S3**. Types of p53 steady state bifurcation curves obtained as kinetic parameters *k_0,basal_* and *j_5_* are varied simultaneously. *k_0,basal_* is the production rate of active p53 in the absence of DNA damage. *j_5_* is a measure of the binding affinity of p53 towards the promoter site of its target genes. Each point corresponds to a p53 steady state bifurcation curve computed for a particular value of *k_0,basal_* and *j_5_*. 4896 steady state bifurcation curves are computed in total. Legend: denotes monostable steady state curves (no oscillation is obtained); O and indicate bistable steady state curves that manifest early switch (in the latter, initial oscillation amplitudes of spiral nodes cause the early-switch); O and indicate bistable steady state curves that manifest saddle-node switch (the latter exhibits limit cycles at the low-p53 states).
