# Supplementary material for: Oscillations of the p53-Akt Network: Implications on Cell Survival and Death
Source: PLoS One. 2009 Feb 6;4(2):e4407. doi: 10.1371/journal.pone.0004407 (PMC2634840; doi:10.1371/journal.pone.0004407)
Supplement: Figure S4 — (0.08 MB DOC) [file pone.0004407.s005.doc]

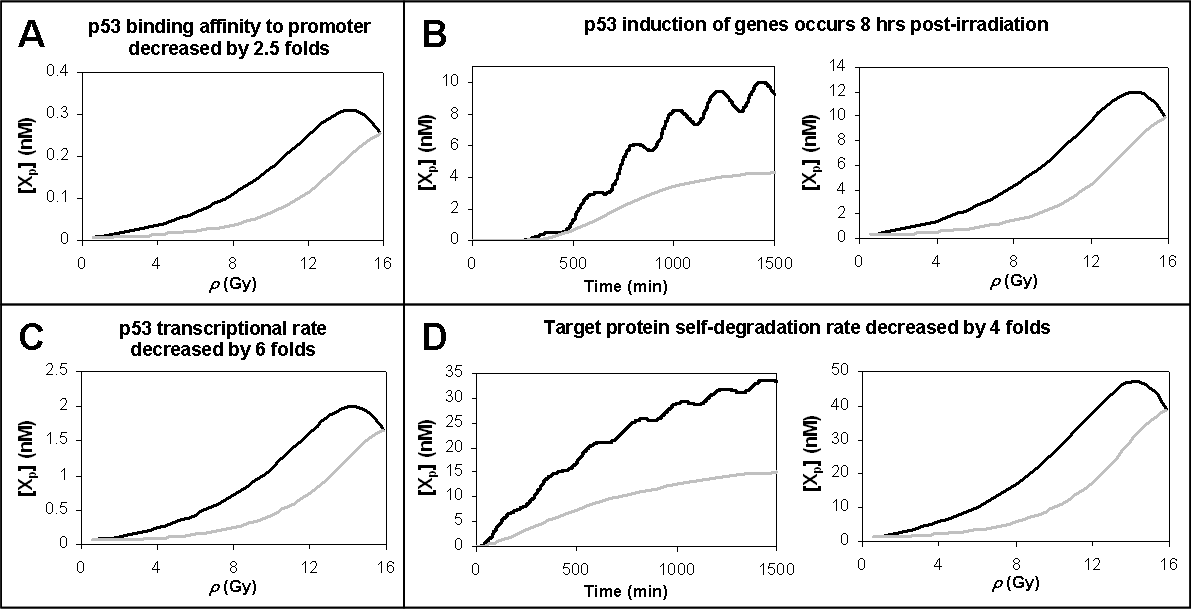


**Figure S4**. Oscillatory p53 induces higher levels of target genes whose expressions have slow dynamics. Steady state level of X_p_ induced by both non-oscillating (gray curve) and oscillating (black curve) p53 for the entire range of *ρ* where limit cycle exists in the *Model*. Kinetic parameter values used in the simulations are identical to those used in Figure 5B (in main text of paper) unless otherwise stated. **(A)** p53 binding affinity to promoter decreased 2.5 fold (*j_11_* = 5 μM). **(B)** p53 induction of genes occurs 8 hrs post-irradiation. A Hill-type function of time (set as 480 min) after irradiation is included as a factor in *v_11_* to simulate an 8 hr time delay for gene expression. Representative time courses of X_p_ expression induced by both non-oscillatory and oscillatory p53 at *ρ * = 12Gy are shown in the left panel. **(C)** p53 transcriptional rate decreased 6 fold (*k_11_* = 0.001 μM/min). **(D)** Target protein self-degradation rate decreased 4 fold. Representative time courses of X_p_ expression induced by both non-oscillatory and oscillatory p53 at *ρ * = 12Gy are shown in the left panel.
