# Supplementary material for: Oscillations of the p53-Akt Network: Implications on Cell Survival and Death
Source: PLoS One. 2009 Feb 6;4(2):e4407. doi: 10.1371/journal.pone.0004407 (PMC2634840; doi:10.1371/journal.pone.0004407)
Supplement: Figure S5 — (0.08 MB DOC) [file pone.0004407.s006.doc]

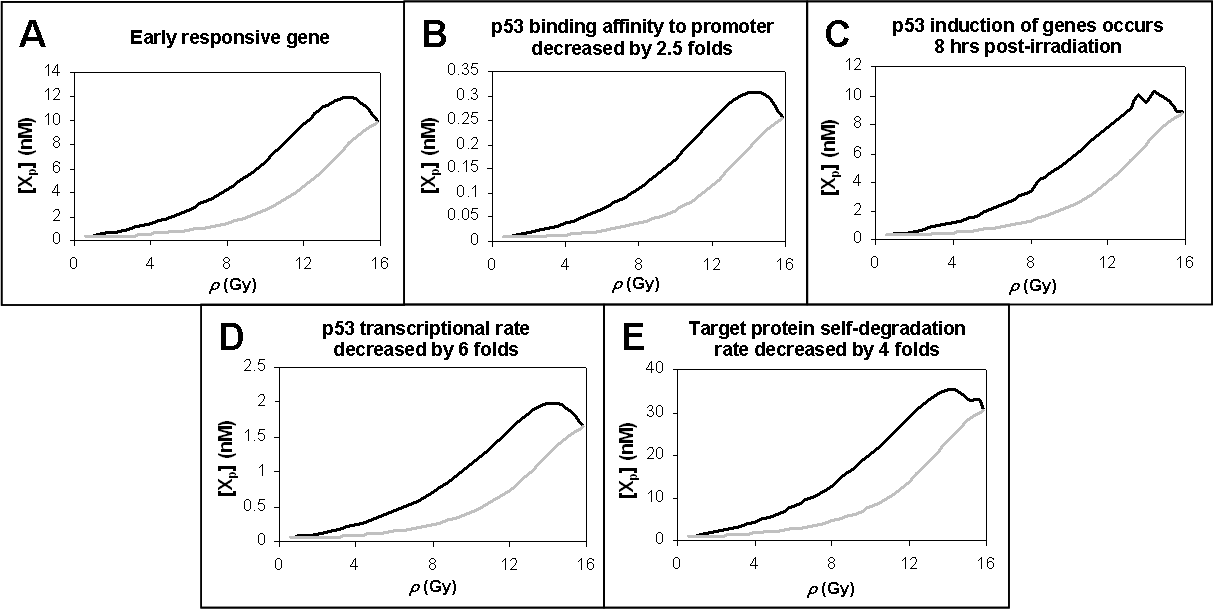


**Figure S5**. Three to four pulses of p53 are sufficient to induce higher level of expressions in both early and late responsive target genes. Level of X_p_ induced at 1200 min (elapsed time for 3 to 4 pulses of p53) by both non-oscillating (gray curve) and oscillating (black curve) p53 for the entire range of *ρ* where limit cycle exists in the *Model*. Kinetic parameter values used in the simulations are identical to those used in Figure 5B (main paper) unless otherwise stated. **(A)** Early responsive gene. Identical parameter values used as in Figure 5B. **(B)** **– (E)** Late/slow responsive genes. **(B)** p53 binding affinity to promoter decreased 2.5 fold (*j_11_* = 5 μM). **(C)** p53 induction of genes occurs 8 hrs post-irradiation. **(D)** p53 transcriptional rate decreased 6 fold (*k_11_* = 0.001 μM/min). **(E)** Target protein self-degradation rate decreased 4 fold.
