# Supplementary material for: Oscillations of the p53-Akt Network: Implications on Cell Survival and Death
Source: PLoS One. 2009 Feb 6;4(2):e4407. doi: 10.1371/journal.pone.0004407 (PMC2634840; doi:10.1371/journal.pone.0004407)
Supplement: Figure S6 — (0.08 MB DOC) [file pone.0004407.s007.doc]

**Figure S6**. Time courses of p53 (red), mdm2 (blue, broken line) and MDM2 (blue) and MDM2*a* (black) in response to *ρ * = 6 Gy.
