# Supplementary material for: Oscillations of the p53-Akt Network: Implications on Cell Survival and Death
Source: PLoS One. 2009 Feb 6;4(2):e4407. doi: 10.1371/journal.pone.0004407 (PMC2634840; doi:10.1371/journal.pone.0004407)
Supplement: Figure S7 — (0.04 MB DOC) [file pone.0004407.s008.doc]

**Figure S7**. Limit cycles generated from the *Model* and a p53-MDM2 model. The standalone p53-MDM2 model is extracted directly from the *Model* depicted in Figure 1B of the main paper by considering only reaction steps involving p53, MDM2 and mdm2; kinetic parameters are identical to those used in the *Model*. The figures show for the range of *ρ* where limit cycles exist in the *Model* (black) and the p53-MDM2 model (gray), the respective oscillation amplitudes of **(A)** p53 and **(B)** MDM2*a*, **(C)** oscillation periods and **(D)** time-delay of MDM2*a* peaks to p53 peaks.
